# Stabilizing synchrony with heterogeneity

**DOI:** 10.1186/1471-2202-16-S1-P270

**Published:** 2015-12-18

**Authors:** Ehsan Bolhasani, Alireza Valizadeh

**Affiliations:** 1 Department of Physics, Institute for Advanced Studies in Basic Sciences, Zanjan, Iran; 2 School of Cognitive Sciences, Institute for Studies in Theoretical Physics and Mathematics, Niavaran, Tehran, Iran

## 

Correlation and synchrony between spike trains of neurons can arise from shared input they receive from other neurons, or from direct connections between the neurons. Physiological heterogeneity can destabilize both coupling-induced and shared input-induced synchronization [1]. In the classical models of synchronization, collective state of a system of coupled oscillators is determined by outcome of rivalry between synchronizing effect of connections and desynchronizing effect of inhomogeneity [2]. Yet there are examples of the systems in which inhomogeneity enhances synchrony [3,4]. In this study we have developed a general framework for the correlation of coupled neuronal oscillators with a given phase sensitivity and we have shown that in some cases, for identical neurons, synchronized state is an unstable attractor and arbitrarily weak noise can destroy synchrony. Inhomogeneity in such systems could stabilize synchrony by providing an asymmetric basin of attraction around the stable phase-locked state. This in turn results in a sharper PDF for the time difference between spikes of the two neurons in presence of noise (see Figure 1A). The analytic results are obtained by solving Fokker-Planck equations for phase oscillators and it is also shown that in presence of stochastic inputs, the most probable phase difference between spike times of the two neurons does not coincide with the stable point of the deterministic equations. Numerical tests on LIF neurons confirm the positive role of inhomogeneity in stabilizing synchrony and increasing correlation of spike trains. The only difference is that for the model neurons with biologically realistic phase response curve (PRC), the time difference between the spikes of two neurons in the stable state increases with inhomogeneity, in the case of LIF neurons, they lock in almost zero phase lag for sufficiently small values of inhomogeneity (see Figure 1B).

**Figure 1:**
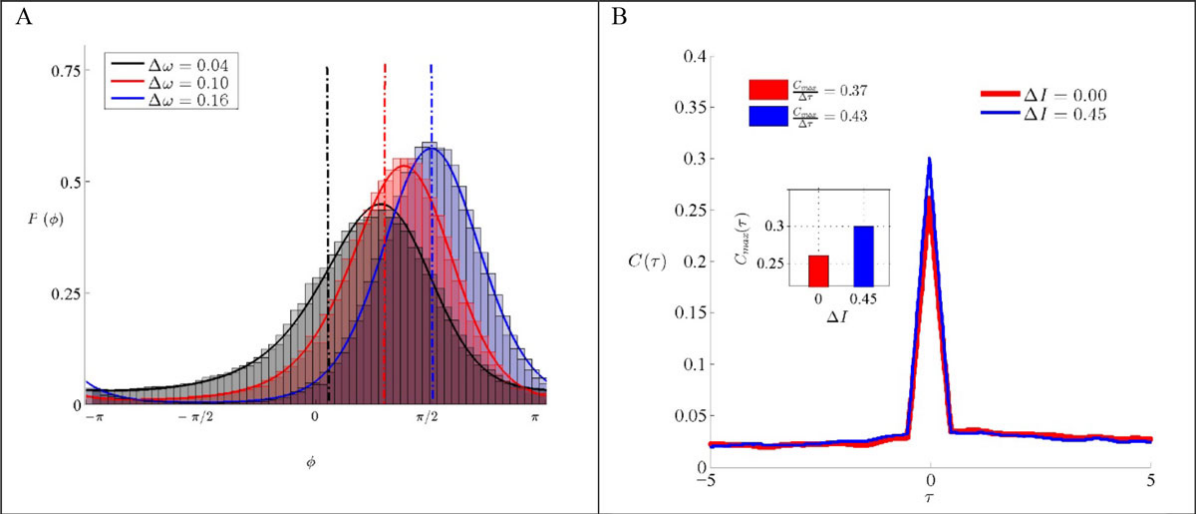
**(A) PDF for the phase difference of two type1 phase oscillators in the presence of noise for three levels of frequency mismatch**. Theoretical predictions (solid lines) are in accordance with numerical results shown as histograms. Dash lines show the phase-locked states in the absence of noise. (B) Cross-Correlogram for two levels of current mismatch for LIF neuron model. Inset: the magnitude of maximum correlation for two neurons shows the positive effect of mismatch in enhancement of correlation.

## Conclusion

While usually physiological heterogeneity is considered as a destructive factor for the synchrony of spike trains, we have shown that correlation of spike trains of two coupled neurons can be enhanced by the mismatch of intrinsic firing rates of the neurons. The result, obtained from analytic solution of pulse-coupled phase oscillators, is valid for all type-I neuronal oscillators with strictly positive phase reset curves, including LIF neurons.
